# Stat3 phosphorylation is required for embryonic stem cells ground state maintenance in 2i culture media

**DOI:** 10.18632/oncotarget.16112

**Published:** 2017-03-10

**Authors:** Dan Wang, Hui Sang, Kaiyue Zhang, Yan Nie, Shuang Zhao, Yan Zhang, Ningning He, Yuebing Wang, Yang Xu, Xiaoyan Xie, Zongjin Li, Na Liu

**Affiliations:** ^1^ Key Laboratory of Bioactive Materials, Ministry of Education, College of Life Sciences, Nankai University, Tianjin, China; ^2^ School of Medicine, Nankai University, Tianjin, China; ^3^ College of Life Sciences, Nankai University, Tianjin, China; ^4^ Stem Cells and Regenerative Medicine Lab, Beijing Institute of Transfusion Medicine, Beijing, China

**Keywords:** embryonic stem cell, ground state, Stat3 signal pathway, imaging

## Abstract

Embryonic stem cells (ES cells) can be maintained its undifferentiated state with feeder cells or LIF, which can activate Jak/Stat3 pathway. Recently, it has been reported a new culture condition comprising serum-free medium with ERK and GSK3β inhibitors (2i) could drive ES cells into a state of pluripotency more like inner cell mass (ICM) in mouse blastocysts called ground state. However, although 2i could sustain ES cells self-renewal, LIF is routinely added. The roles of Stat3 activation are still unclear now. Here we investigated whether Jak/Stat3 might also contribute to the induction of ground state pluripotency. We introduced a lentiviral construct with 7-repeat Stat3-binding sequence to drive Renilla luciferase into ES cells, which can be used as a reporter to detect Stat3 activation by noninvasive bioluminescence imaging. Using this ES cells, we investigated the role of Stat3 activation in ground state maintenance. The results showed that Stat3 could be activated by 2i. Stattic, a chemical inhibitor of Stat3 phosphorylation, could effectively inhibit Stat3 activation in ES cells. When Stat3 activation was suppressed, ground state related genes were down regulated, and ES cells could not be maintained the ground state pluripotency even in 2i medium. All of these results indicate Stat3 activation is required in ground state maintenance.

## INTRODUCTION

Mouse embryonic stem cells (ES cells) are derived from inner cell mass (ICM) of blastocyst embryos, which possess the ability of self-renewal and differentiation into any cell type of the three-germ layers [1]. ES cells sustain the property by a number of key transcription factors of cytokine-responsive genes, such as Oct4, Nanog and Sox2 [2–4]. Only the optimal medium contents for the activation of signaling pathways could ES cells maintain the pluripotency *in vitro*.

The role of leukemia inhibitory factor (LIF) in maintaining the pluripotency and self-renewal of mouse ES cells has been reported [5], and the biochemical function of LIF demonstrated a requirement for activation of the transcriptional factor signal transducer and activator of transcription 3 (Stat3) [6]. LIF binds to the cell surface LIF receptor (LIFR), causing the heterodimer formation of LIFR and the signal transducer glycoprotein 130 (gp130), which activates gp130-associated kinase (Jak) and phosphorylates the tyrosine residues in the gp130 cytoplasmic domain. Subsequently, the phosphotyrosine serves as the docking site to recruit the Src-homology-2 (SH2) domain containing Stat3, which could be phosphorylated by activated Jak at tyrosine 705 (Y705). Following phosphorylation, the Stat3 proteins form dimmers enter the cell nucleus to bind response elements and regulate the expression of the target genes [7, 8].

Recently, with the development of single cell technology, more and more evidences showed that pluripotent cell populations in ES cells display significant heterogeneity at the molecular level [9, 10], like pluripotency markers Nanog [11], Tbx3 [12] and Rex1 [13]. It has been found that blockade of MEK pathway and suppression of glycogen synthase kinase-3β (GSK3β) could sustain ES cells self-renewal and drive ES cells into naïve state of pluripotency [14, 15]. Naïve state, also described as ground state, reflects the pluripotency state of ICM in the preimplantation embryo and shows little evidence of the expression of lineage markers. Ground state ES cells could be maintained by blocking inductive differentiation pathways using PD0325901 and CHIR99021, which respectively inhibit the activation of ERK and GSK3β [16–18]. By contrast, epiblast stem cells (EpiSCs) derive from the late epiblast of the mouse post-implantation embryo and robustly differentiate into the major somatic cell types as well as primordial germ cells [19]. EpiSCs differs from naïve ES cells in various respects such as cellular morphology, gene expression profile and epigenetic modification. Unlike ES cells, EpiSCs depend on ActivinA and Fgf2 to maintain the pluripotency instead of Jak/Stat3 pathway, and they display a restricted developmental potency, which couldn't produce chimeras. EpiSCs express early lineage markers such as Brachyury (T), which is a characteristic feature of primed state. It has been reported that EpiSCs could be passaged and cultured in N2B27 medium with ActivinA/FGF2 *in vitro* [20]. Furthermore, naïve state ES cells can be converted into EpiSCs by withdrawal of LIF and culture in ActivinA/FGF2 [21, 22], and conversely, EpiSCs can be reprogrammed to ground state pluripotency if ES cells are cultured in 2i/LIF medium [14].

Although 2i could sustain ES cells ground state pluripotency without the activation of Jak/Stat3 pathway, LIF is required in addition. Furthermore, several groups have reported the activation of Stat3 could facilitate the maintenance of ES cells in ground state [23, 24]. However, it is still unclear about the relationship between Stat3 activation and 2i condition in ES cells ground state maintenance. Here we used a visible reporter system to monitor Stat3 activation and investigated whether Jak/Stat3 might also contribute to induction of ground state pluripotency in 2i culture medium.

## RESULTS

### Construction of Stat3-Rluc-GFP-reporter ES cells

**Figure 1:**
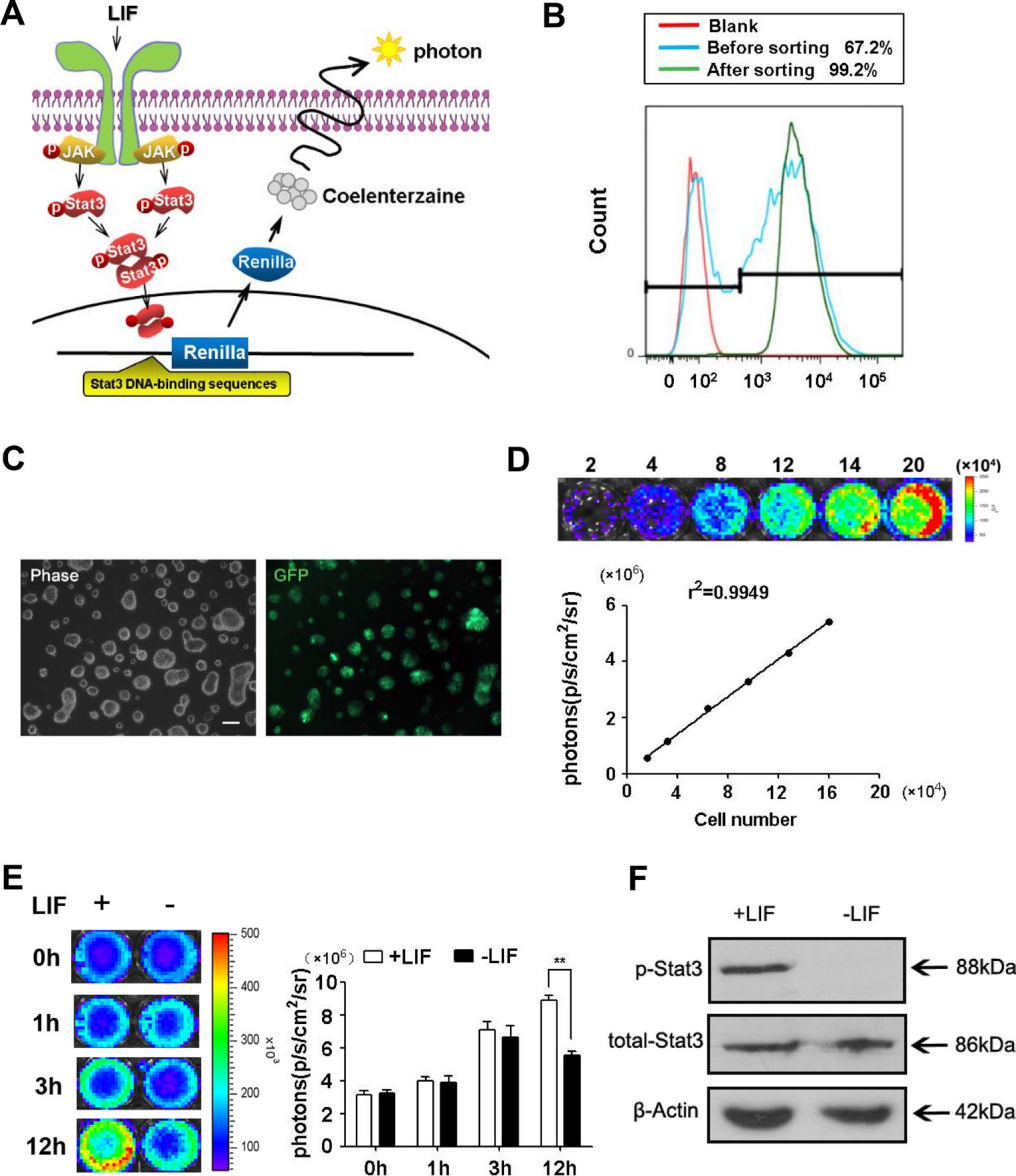
Construction of Stat3-Rluc-GFP-reporter ES cells (**A**) Schematic for noninvasive imaging of Stat3 activity. Jak kinases were phosphorylated by cell surface receptor tyrosine kinases in response to cytokine activation. Jak kinases could phosphorylate Stat3, which then transfer into nuclear and bind to the Stat3 DNA-binding sequence to activate the reporter. (**B**) Detecting the GFP positive cell percentage in total transfection cells and in sorted stable cells using flow cytometry. The transfection efficiency was 67.2%. 99.2% of cells are GFP positive cells after sorting. (**C**). Transduced ES cells are strongly positive for GFP on fluorescence microscopy. Scale bars: 100 μm. (**D**). Imaging analysis of stably transduced ES cells shows a strong correlation between cell numbers and Fluc reporter gene activity. (**E**) Renilla luciferase imaging showed bioluminescence signals at different time points with LIF present or not. Histogram showed the quantitative analysis of imaging signals. (*n* = 3; *p <.05, **p < .01, ***p <.001). (**F**). LIF activated Stat3 phosphorylation in ES cells. Representative Western blots showing the level of p-Stat3 and total Stat3. The figure shows representative data from three independent experiments.

To evaluate the role of Stat3 activation in regulating ES cells pluripotency, we used a phoshphor-Stat3 (p-Stat3) reporter gene to monitor the activation of Stat3. The reporter construct carried by a self-inactivating lentiviral vector capable of stably transduced ES cells. In addition, the reporter comprised a 7-repeat of Stat3-recognition sites (enhancer) and a small TA promoter, the design of tandem repetition could improve the expression of reporter gene significantly and Renilla luciferase could be used for quantitative bioluminescence imaging technique *in vitro*. The constitutive CMV promoter driving GFP as a selection marker allowed us to sort the stably transduced clones by fluorescence-activated cell sorting (FACS). LIF could bind the cell surface receptor, which phosphorylates Jak kinase and subsequently activates Stat3. Once activation, Stat3 proteins form dimmers and enter the cell nucleus to bind response elements, which induced the expression of Renilla luciferase. The substrate coelenterazine could be catalyzed by Renilla luciferase and produce photons, the number of photons could be detected by CCD camera (Figure 1A).

To develop an imaging approach, the Stat3 reporter vector was transfected into ES cells using the lentiviral infection, designated as pStat3-D3 ES cells. The lentiviral transduction of ES cells showed a high efficiency (67.2%) based on the flow cytometry analysis with a FITC (530 ± 15 nm) filter setting (Figure 1B). GFP positive ES cells were then isolated from the total cells using FACS. The stable clones were next confirmed by GFP expression using fluorescence microscopy (Figure 1C), and the relatively stable activities remained for 20 passages over 6 weeks (data not shown). We also examined control wild-type D3 (wt-D3) ES cells and pStat3-D3 ES cells proliferation at indicated time points (day 0, day 2, day 4 and day 6) and observed no significant changes between the two populations (Supplementary Figure 1). To confirm the level of reporter gene activities correlated with the cell numbers, ES cells (2 × 10^4^ to 2 × 10^5^) were analyzed using imaging and cell counting respectively. Overall, there was a robust relationship between cell number and Renilla luciferase activity (r^2^ = 0.9949) (Figure 1D).

AS we all known, Jak/Stat3 pathway plays a fundamental role in promoting the pluripotency establishment and the pathway could be activated by cytokine such as LIF [25]. To assess the temporal response of p-Stat3, the pStat3-D3 ES cells were starved of LIF for 12 hours and then stimulated with LIF (1000 units/mL). Renilla luciferase imaging showed the Stat3 could be phosphorylated by LIF significantly in 12 hours after LIF treatment (Figure 1E). Western blot analysis of total Stat3 and p-Stat3 also confirmed that Stat3 was activated treated with LIF for 12 hours. Without LIF, Stat3 phosphorylation was almost undetectable (Figure 1F), which was consistent with imaging results. All of these suggested that Stat3 activation could be monitored by imaging method, which was more convenient compared with western blot.

### 2i/LIF culture media drives ES cells into ground state

**Figure 2:**
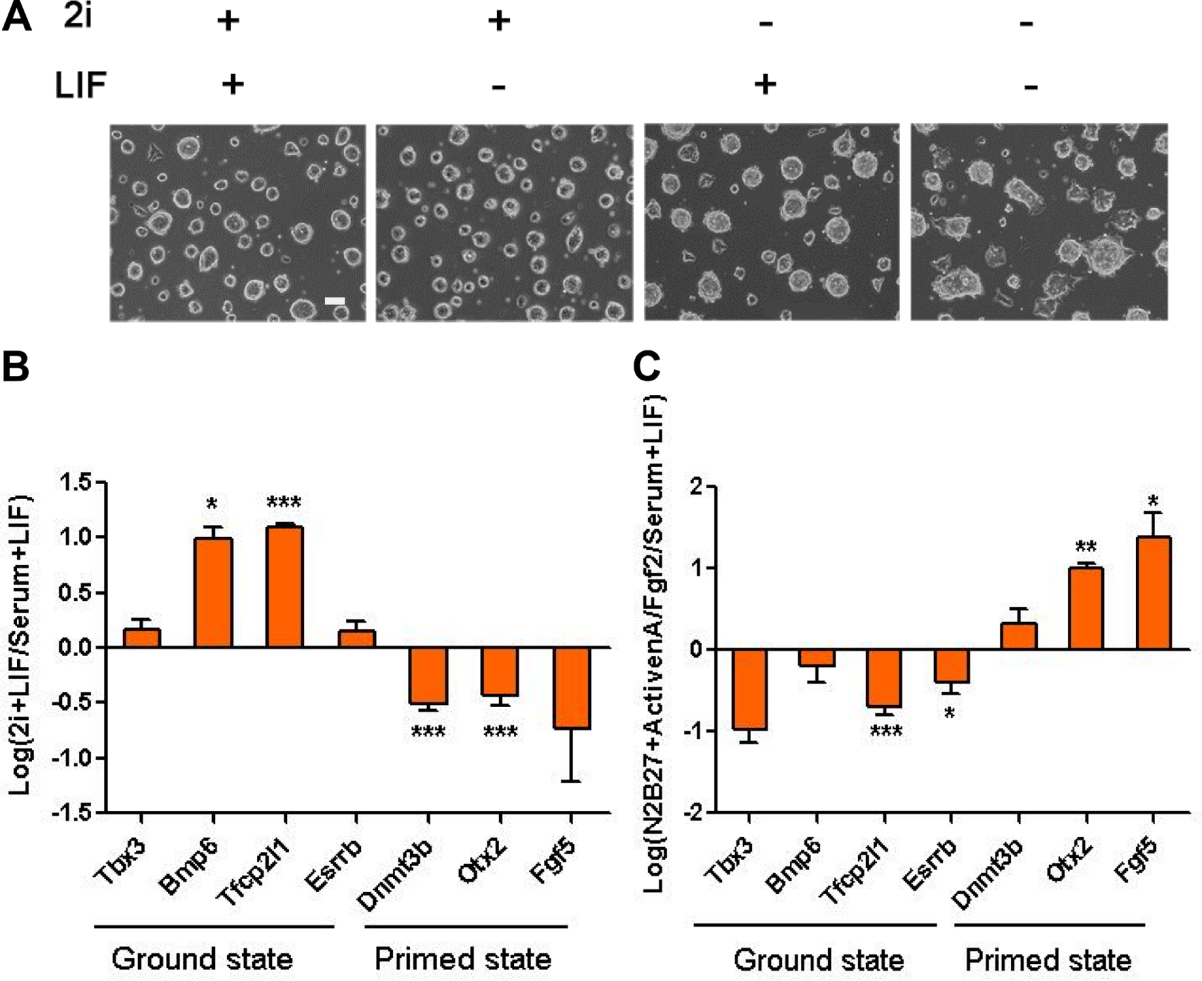
2i/LIF medium could sustain the ground state of ES cells while ActivinA/FGF2 passage the ES cells into primed state (**A**) Phase contrast images showed the cell morphology of pStat3-D3 cells in 2i+LIF, 2i-LIF, serum+LIF and serum-LIF. Images were taken after 48 hours in different culture conditions. Scale bars: 100 μm. (**B**) Relative expression of ground state genes (*Tbx3, Bmp7, Tfcp21l and Esrrb*) and primer state genes *(Dnmt3b, Otx2 and Fgf5)* in pStat3-D3 cells after 48 hours of 2i /LIF. (*n* = 3; *p <.05, **p < .01, ***p < .001). (**C**) Relative expression of ground state genes (*Tbx3, Bmp7, Tfcp21l and Esrrb*) and primer state genes (*Dnmt3b, Otx2 and Fgf5*) in pStat3-D3 cells after 48 hours of ActivinA/FGF2. (*n* = 3; *p < .05, **p < .01, ***p < .001).

It has been reported that the activation of Stat3 inducted by LIF could translocate into nucleus, where it regulated transcription downstream target genes to maintain the identity of ES cells [26]. However, ES cells cultured in serum/LIF are heterogeneous. 2i/LIF could maintain ES cells morphologically uniform and rather homogeneous in expression of ground pluripotency regulators [27]. We cultured pStat3-D3 ES cells for 48 hours in four different conditions (2i+LIF, 2i-LIF, serum+LIF, or serum-LIF). In 2i+LIF, pStat3-D3 cells retained typical ES cells morphology. In contrast, in serum+LIF or serum-LIF, pStat3-D3 cells differentiated within two days and appear to be flat in colony morphology (Figure 2A). Real-time PCR analysis confirmed the ES cells in 2i+LIF medium showed activated expression of ground pluripotency markers (*Tbx3*, *Bmp7*, *Tfcp2l1*, and *Esrrb*) and suppressed expression of primed state markers such as *Dnmt3b*, *Otx2*, and *Fgf5* (Figure 2B). The culture condition of 2i+LIF has a more significant effect on the maintenance of ground state of ES cells than 2i-LIF (Supplementary Figure 2). Moreover, withdrawing LIF and supplementing the culture medium with ActivinA/ FGF2 resulted in the transition of naive ES cells to primed state ES cells (Figure 2C). These results proved that 2i/LIF medium could maintain pStat3-D3 cells in ground state.

### 2i treatment increases the activity of p-Stat3

**Figure 3:**
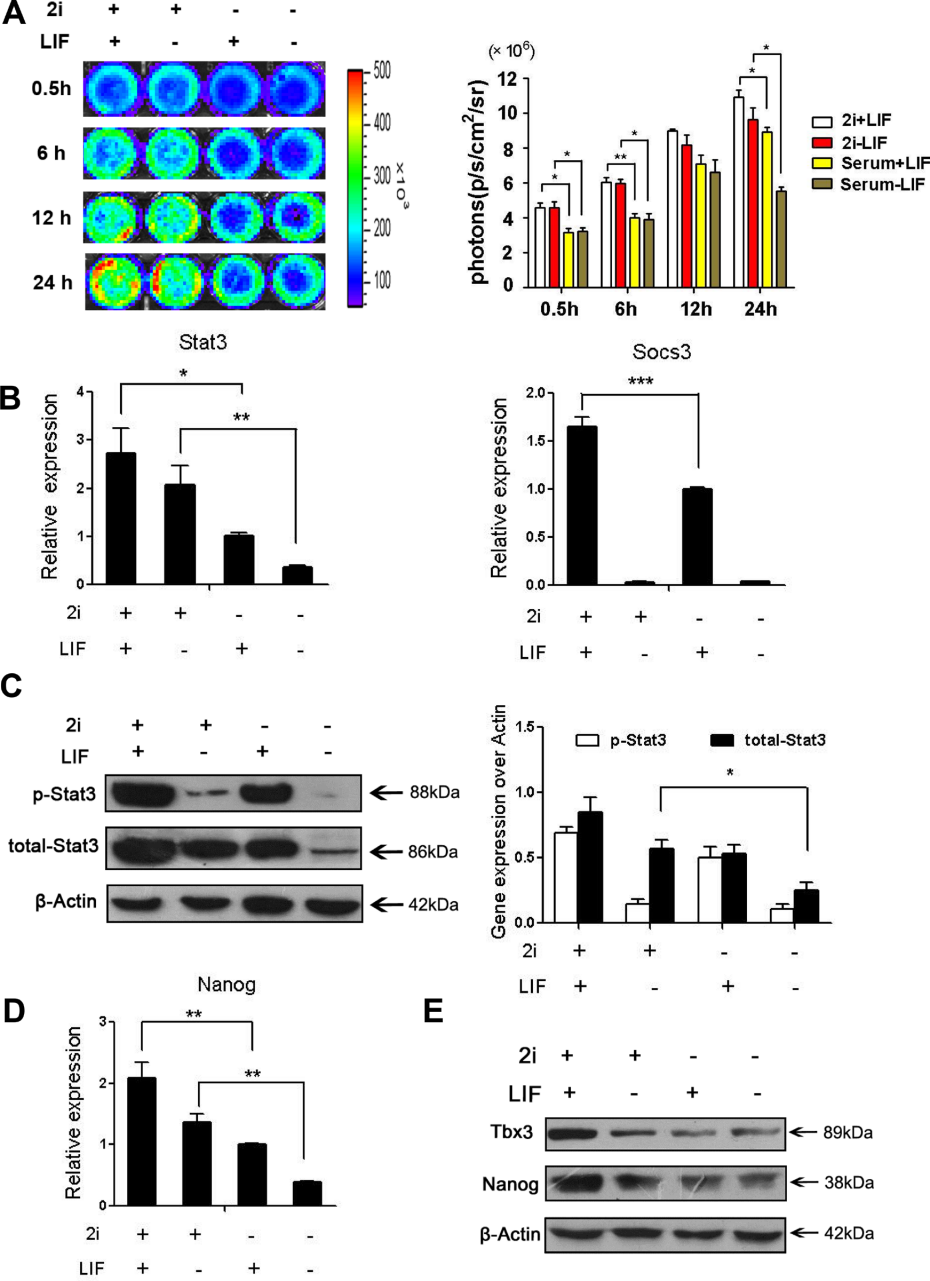
2i treatment increased phoshphor-Stat3 activity and Stat3 activation is limiting for ground state pluripotency (**A**) Renilla luciferase imaging showed reporter gene activity with/without LIF and 2i at different time points. Histogram showed the quantified Renilla luciferase. (*n* = 3; *p <.05, **p <.01, ***p <.001). (**B**) Real-time PCR analysis of *Stat3* and the downstream target gene *Socs3* expression in 2i+LIF, LIF, 2i or none of them. (*n* = 3; *p <.05, **p <.01, ***p <.001). (**C**) Western blot analysis of total Stat3 and p-Stat3 in at different culture conditions. The figure shows representative data from three independent experiments. (**D**) Real-time PCR analysis of ground state gene *Nanog* treated with 2i or LIF for 48 hours. (*n* = 3; *p <.05, **p <.01, ***p <.001). (**E**) Western blot analysis of ground state markers Nanog and Tbx3 after 2i or LIF stimulation for 48 hours. The figure shows representative data from three independent experiments.

It has been reported that two inhibitors (GSK3β inhibitor and MEK kinases inhibitor) can sustain ES cells self-renewal and drive ES cells into ground state pluripotency [14, 19]. As previous report, the activation of Stat3 is also a limiting factor for reprogramming to ground state pluripotency [28]. We used imaging approach to examine the activity of Stat3 in different culture conditions and investigated the role of Stat3 in ES cells ground state maintenance. Renilla luciferase imaging showed that p-Stat3 was increased in 2i with or without LIF after 24 hours treatment (Figure 3A). That means 2i could activate Stat3 signal transduction. We next investigated the expression level of *Socs3*, a downstream target of Stat3. *Socs3* expression level was elevated with 2i/LIF treated for 48 hours (Figure 3B). *Stat3* expression was also increased in 2i+LIF compared with serum+LIF (Figure 3B) in accordance with the result of western blot (Figure 3C). To further investigate the role of Stat3 in ground state pluripotency, we next analyzed the expression of *Nanog*, a ground state marker, which expression was increased after 2i+LIF treatment compared with serum+LIF (Figure 3D). Western blot also confirmed the ground state maintenance by detecting the expression of Tbx3 and Nanog (Figure 3E). These results indicated that Stat3 signal pathway was activated in ground state pluripotency.

### Stat3 inhibition facilitated ES cell conversion from ground state to primed state

**Figure 4:**
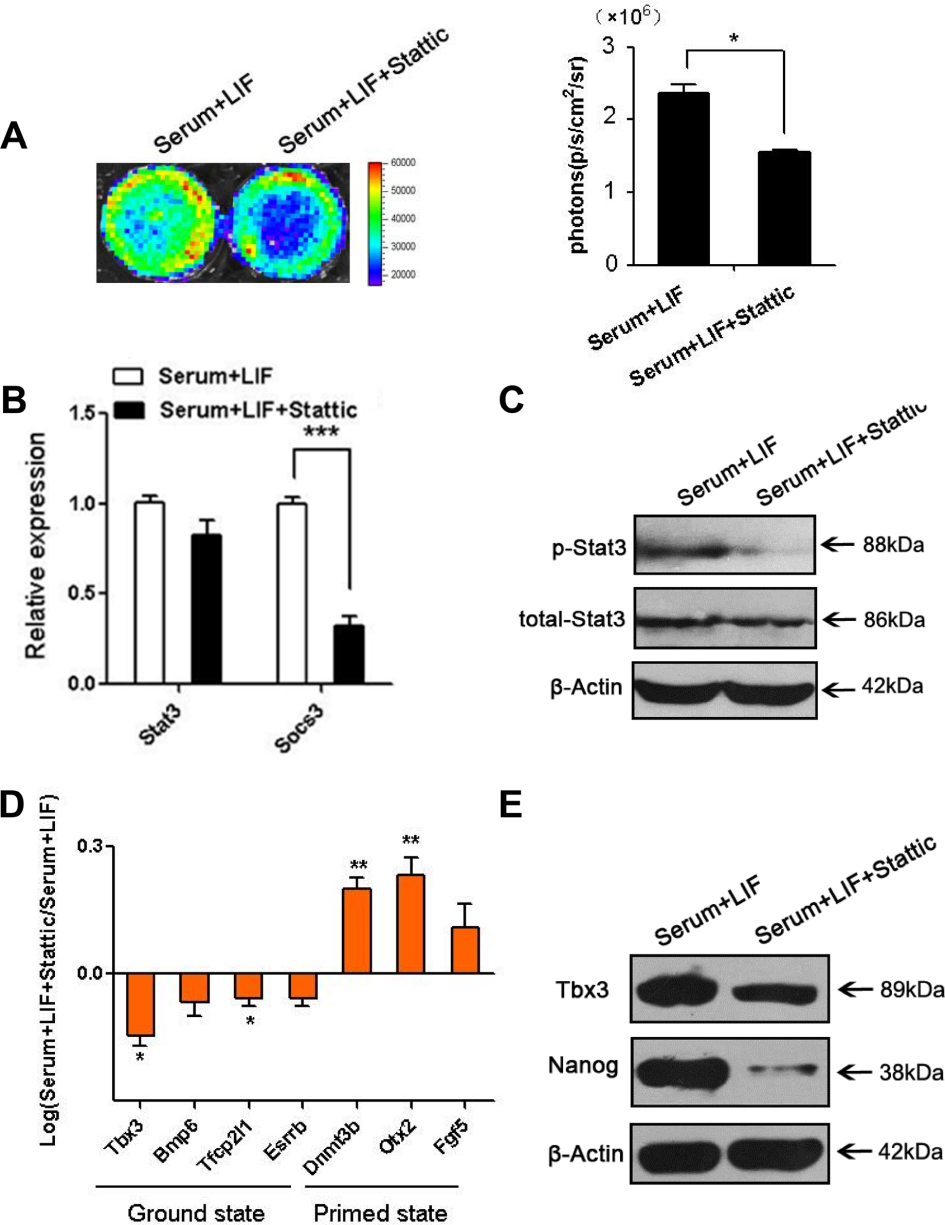
Stattic repressed the phosphorylation of Stat3 and relative gene expression (**A**) Renilla luciferase imaging showed p-Stat3 with LIF and Stattic after 48 hours. Histogram showed the quantified Renilla luciferase activity. (*n* = 3; *p < .05, **p < .01, ***p < .001). (**B**) Real-time PCR analysis of *Stat3* and the downstream target gene *Socs3* with Stattic (p-Stat3 inhibitor) treatment. Images were taken after 48 hours in LIF and Stattic. (*n* = 3; *p < .05, **p < .01, ***p < .001). (**C**) Western blot analysis of total Stat3 and p-Stat3 after Stattic stimulation for 48 hours. The figure shows representative data from three independent experiments. (**D**) Real-time PCR analysis of ground state genes *(Tbx3, Bmp7, Tfcp21l and Esrrb*) and primed state genes (*Dnmt3b, Otx2 and Fgf5*) after Stattic treatment for 48 hours. (*n* = 3; *p < .05, **p < .01, ***p < .001). (**E**) Western blot analysis of ground state marker Nanog and Tbx3 after Stattic stimulation for 48 hours. The figure shows representative data from three independent experiments.

All above results suggested that 2i could activate Stat3 activation. We next investigated whether the activation of Stat3 was necessary for ES cells ground state maintenance in 2i/LIF media. Stattic, a small molecule inhibitor of Stat3, selectively inhibits activation, dimerization, and nuclear translocation of Stat3 [29]. To analyze the function of Stat3 inhibition in ES cells, we treated pState-D3 cells with Stattic at different concentrations for 24 hours or 48 hours. As presented in Supplementary Figure 3A, most ES cells died at concentration of 10 or 15 μM Stattic for 48 hours. As judged by western blot analysis using phospho-specific antibodies against Stat3 tyrosine705 residue, Stattic effectively blocked Stat3 phosphorylation induced by LIF at 8 μM culturing for 48 hours (Supplementary Figure 3B). Renilla luciferase activities displayed Stat3 phosphorylation was repressed with Stattic (Figure 4A), which were confirmed using real-time PCR (Figure 4B) and western blot (Figure 4C). Then we analyzed several genes expression related with ground state and primed state after Stattic treatment. pStat3-D3 cells were sensitive to Stattic and the ground state genes (*Tbx3*, *Bmp7*, *Tfcp21l*, and *Esrrb*) were downregulated, whereas the primed state genes (*Dnmt3b*, *Otx2*, and *Fgf5*) were upregulated with serum+LIF+Stattic treatment compared with serum+LIF (Figure 4D). Nanog and Tbx3 (ground state marker) protein level was significantly decreased with Stattic treatment (Figure 4E). All the data suggested the importance role of Stat3 in ES cells ground state pluripotency maintenance. Stat3 signaling inhibition promoted ES cells transition into primed state.

### Stat3 signal pathway activation was required for the maintenance of ground state

**Figure 5:**
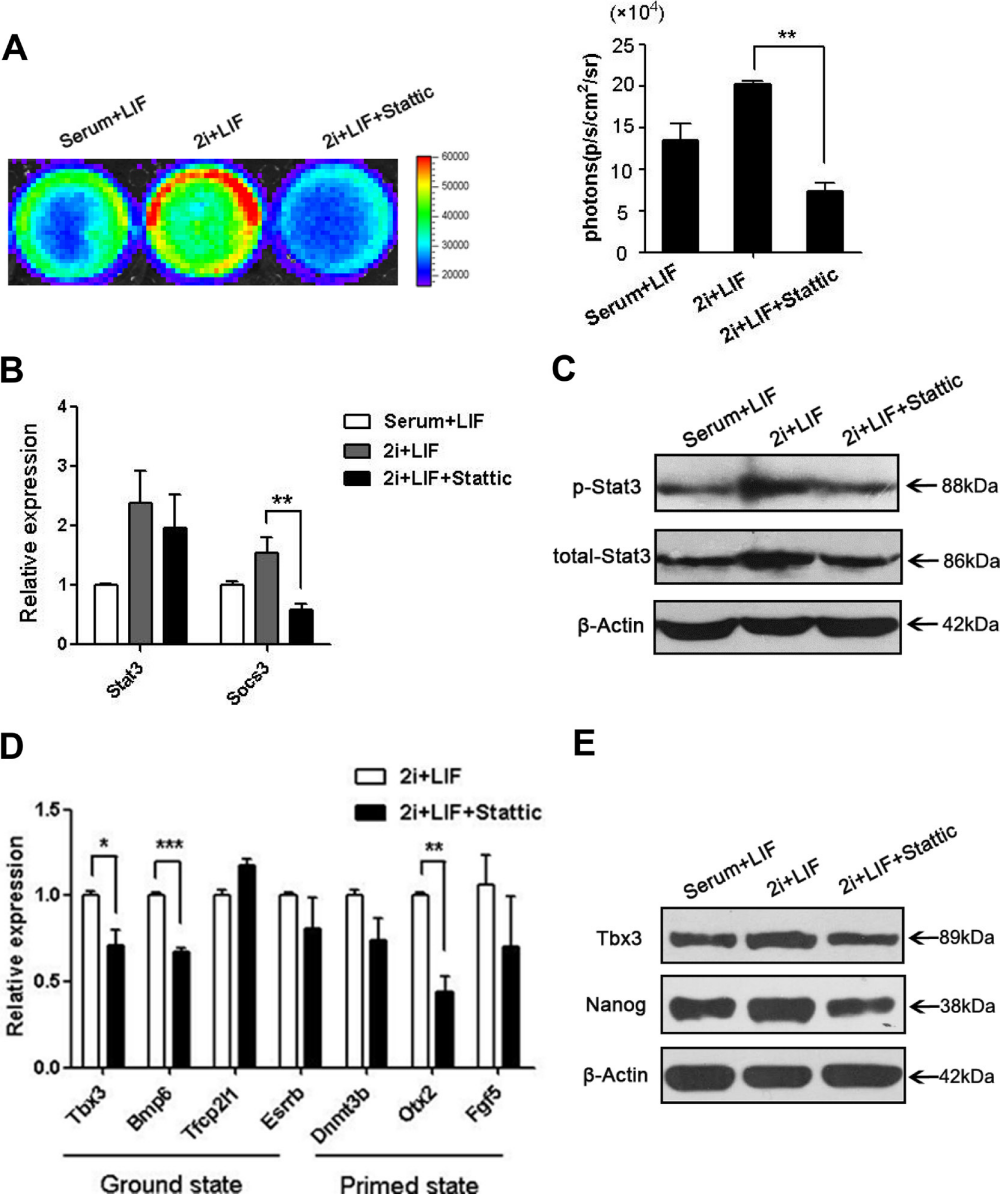
p-Stat3 is a limited factor for the ground state of ES cells culturing in 2i+LIF (**A**) Renilla luciferase imaging showed reporter gene activity in serum+LIF, 2i+LIF and 2i+LIF+Stattic. Histogram showed the quantified Renilla luciferase activity. (*n* = 3; *p < .05, **p < .01, ***p < .001). (**B**) Real-time PCR analysis of *Stat3* and the downstream target gene *Socs3* with 2i+LIF+Stattic treatment. Images were taken after 48 hours in LIF and Stattic. (*n* = 3; *p < .05, **p < .01, ***p < .001). (**C**) Western blot analysis of Stat3 and p-Stat3 with 2i/LIF plus inhibitor treatment. The figure shows representative data from three independent experiments. (**D**) Real-time PCR analysis of ground state genes (*Tbx3, Bmp7, Tfcp21l and Esrrb*) and primed state genes *(Dnmt3b, Otx2, and Fgf5*) after 2i+LIF+Stattic treatment for 48 hours. (*n* = 3; *p < .05, **p < .01, ***p < .001). (**E**) Western blot analysis of ground state marker Nanog and Tbx3 after 2i+LIF+Stattic stimulation for 48 hours. The figure shows representative data from three independent experiments.

To further evaluate whether p-Stat3 is necessary to the maintenance of ground state mediating by 2i, we cultured pStat3-D3 ES cells in 2i+LIF+Stattic for 48 hours to block the activation of Stat3. Renilla luciferase imaging showed a significant inhibition of Stat3 phosphorylation when ES cells were treated with 2i/LIF plus Stattic (Figure 5A), which was consisted with the result of western bolt (Figure 5C). The expression *Socs3* was considerably decreased when ES cells treated with Stattic, further demonstrating the inhibitor efficiently suppressed the activation of Stat3 (Figure 5B). Then we detected the ES cells pluripotency state by analyzing the ground/primed state genes expression. Ground state related genes expression were decreased when p-Stat3 was inhibited (Figure 5D), showing 2i media without Stat3 activation couldn't sustain ES cells ground states, in accordance with the result of Nanog and Tbx3 at protein level (Figure 5E). All of these results showed when Jak/Stat3 pathway was inhibited ES cells could not be maintained in ground state even with 2i presence. Thus, we got the conclusion that Stat3 activation was necessary for ES cells ground state maintenance.

## DISCUSSION

ES cells grown in serum/LIF contained two cell subpopulations based on the expression of Nanog, Rex1, and other pluripotency factors [10, 13]. One subpopulation has a similar gene expression signature like the ICM called ground state ES cells. Another subpopulation termed primed state ES cells which like EpiSCs, which has high expression level of early lineage markers such as Brachyury (T) and Ffg5. Different from LIF is necessary for ES cells pluripotency maintenance, FGF2 and ActivinA were required for primed ES cells and EpiSCs self-renewal [21, 22]. Under 2i/LIF culture condition, the two populations collapsed into ground state and displayed uniform in morphological and in the expression of naïve pluripotency regulators [14, 30]. Jak/Stat3 pathway has been identified as the core signaling pathway for ES cells pluripotency maintenance [31]. Recently several reports showed the activation of Stat3 could induce the incompletely reprogrammed cells back to ground state pluripotency [28]. Van Oosten *et al* showed activation of FGF-ERK signaling couldn't induce ES cells to primed state when Stat3 was activated. Even in ActivinA/FGF2 media (the primed state culture condition), Jak/Stat3 pathway activation could facilitate the conversion from primed state to naïve state [23].

2i could sustain ES cells in ground state pluripotency, in which LIF is always required. Here we demonstrated whether the maintenance of ground state mediating by 2i was involved in the activation of Stat3. We introduced a lentiviral construct with 7 repeat Stat3-binding sequence driving Renilla luciferase into ES cells, which could acquire the temporal kinetics of Stat3 activity during the activation of Jak/Stat3 pathway without disrupting the native cell structure [32]. Our results indicated 2i/LIF medium could maintain the self-renewal phenotype of ES cells and drive ES cells into a state of ground state pluripotency. In addition, 2i treatment increased the activity of p-Stat3 activity significantly with western bolt, real-time PCR and bioluminescence imaging analyses. As previous studies reported, Stat3 activation is limited for ground state pluripotency [28]. In this study, we further evaluated p-Stat3 is required for the maintenance of ground state mediating by 2i by using the inhibitor of p-Stat3, which could efficiently block the phosphorylation of Stat3 Tyr705. We cultured ES cells in 2i+LIF+Stattic and serum+LIF+Stattic respectively for 48 hours, and then analyzed the ES cells pluripotency carefully. As expected, in serum+LIF culture media, Stat3 inhibition facilitated ES cells conversion into the primed state pluripotency. When Stat3 pathway was suppressed, ES cells could not maintained in ground state even in 2i medium. All of the above results suggested the important role of Stat3 signal pathway in ES cells ground state pluripotency maintenance.

In this paper, the application of reporter gene construct and bioluminescence imaging give us a good tool to observe the activation of p-Stat3 temporally, quantitatively and noninvasively compared to the traditional experiment methods. Molecular imaging could be used in further study of the mechanism about how Stat3 played the roles in the maintenance of ground state of ES cells.

## MATERIALS AND METHODS

### ES cells culture

The mouse D3-ES cell lines were grown on plates pre-coated with 0.1% gelatin and culturing in DMEM medium (Corning) supplemented with 15% FBS (Hyclone), 1% L-Glutamine (Corning), 1% NEAA (Gibco), 1% penicillin/streptomycin (Gibco), 1% β-mercaptoethanol (Sigma), and 1000 units/ml of LIF (Millipore) at 37°C in a 5% CO_2_ humidified atmosphere. Stat3-Rluc-GFP-reporter ES cells (pStat3-D3 cells) were grown in N2B27 (Life technologies) medium supplemented with 3 uM CHIR99021 (Sigma), 1uM PD0325901 (Sigma) and 1000 units/ml of LIF to drive ES cells into ground state as preciously described [24]. ES cells in primed state were cultured in N2B27 medium with ActivinA (20 ng/ml, PeproTech) and FGF2 (12 ng/ml, PeproTech). The inhibitor Stattic (Selleck) was used at concentration of 8μM for 48 hours, which inhibits the phoshphor-Stat3 Tyr705 of ES cells [33].

### Lentivirus package and transfection

Human embryonic kidney 293T cells were cultured in DMEM medium with 10% FBS, 1% L-Glutamine and 1% Penicillin/Streptomycin and used to produce lentiviral vectors for stable cell line generation. Briefly, 293T cells were grown in 6-well gelatin coated plates and then the Stat3 reporter vector was co-transfected with the packaging vector psPAX2 and Pmd2.G into 293T cells by Lipofectamine2000 (Invitrogen) [25]. After 24 hours, change media to fresh ES culture media and collect viruses by removing the cell media at 48 h after transfection. For lentiviral infection, ES cells were seeded into 6-well plates and incubated with lentivirus and 3μl/mL polybrene (Sigma). The lentivirus solution was replaced with regular media 24h later. Then, GFP positive cells were processed for FACS sorting using a FACS Diva (BD Biosciense).

### ES cell proliferation assay

After lentiviral transfection, control D3-ES cells and pStat3-D3 cells were plated uniformly in 6-well plates at a density of 2 × 10^5^ cells per well. The cell number of ES cells was measured at indicated time points (day 0, day 2, day 4 and day 6).

### Bioluminescence imaging of reporter gene expression

Renilla luciferase activity was measured by bioluminescence imaging. In sequential noninvasive imaging, ES cells were exposed to 1.5 μg/mL of coelenterazine directly supplemented in the medium and detected with a cooled charge-coupled device (CCD) bioluminescence camera (*In Vivo* Imaging System, IVIS 50; Xenogen, Alameda, CA) immediately. The gray scale photographic images of bioluminescence emitted from the ES cells were super imposed and analyzed with Living Image software. Photon emission was acquired for 3 min and bioluminescence was quantified in units of maximum photons per second per centimeter square per steradian (p/s/cm2/sr) as previously described [34].

### Real-time PCR

TRIzol reagent (Invitrogen) was used to extract total RNA following the manufacturer's instructions. cDNA was synthesized with oligo (dT) as primers, using the First-strand cDNA Synthesis System (TransGen Biotech). The mRNA expression levels were quantified using TransStart Green qPCR SuperMix Kit (TransGen Biotech). Average threshold cycle (Ct) values from the triplicate PCR reactions for a gene of interest (GOI) were normalized against the average Ct values for *Actin* from the same cDNA sample. The 2^−ΔΔCt^ method was used to determine the relative mRNA folding changes. Real-time PCR analysis was performed on Opticon^®^ System (Bio-Rad, Hercules, CA), using the primers shown in Supplementary Table 1.

### Western blot

For protein analysis, ES cells were washed three times with PBS, and then were lysed with RIPA lysis buffer (Cowei) in the culture wells. Cell debris was removed by centrifugation. Protein concentration was determined by using the BCA Protein Assay kit (Thermo). Protein was collected and was separated by 10% SDS polyacrylamide gel electrophoresis and then transfer to PVDF membrane. The blots were probed with primary antibodies overnight at 4°C, and then for 1.5 hours at room temperature with appropriate secondary antibodies. Pierce ECL Western Blotting Substrate (Millipore) was used to detect the signal. The primary antibodies used include Stat3 (1:1000, Santa Cruz), p-Stat3-Y705 (1:1000, Abcam), Tbx3 (1:1000, Abcam), β-Actin (1:1000, Biotechnology), H3.1 (1:1000, Sungene), and Nanog (1:1000, Bethyl).

### Statistical analysis

All experiments were repeated at least three times and each condition was analyzed in triplicate. Data are expressed as mean-standard error of measurement (SEM). For statistical analysis, two-tailed Student's *t*-tests were performed. Differences were considered statistically significant at *p* ≤ 0.05.

## SUPPLEMENTARY MATERIALS FIGURES AND TABLES


