# Supplementary material for: Stat3 phosphorylation is required for embryonic stem cells ground state maintenance in 2i culture media
Source: Oncotarget. 2017 Mar 10;8(19):31227–37. doi: 10.18632/oncotarget.16112 (PMC5458203; doi:10.18632/oncotarget.16112)
Supplement: Supplementary file 1 [file oncotarget-08-31227-s001.pdf]

Stat3 phosphorylation is required for ES cells ground state maintenance in 2i culture media

Supplementary Materials

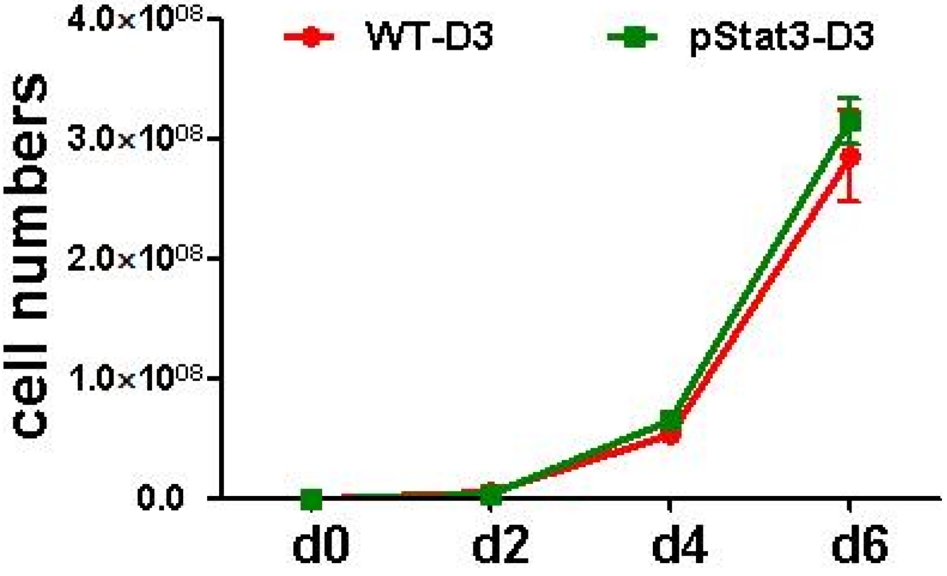

Supplementary Figure 1: Cell proliferation assay between pStat3-D3 and control ES. Growth curves showed no significant difference between wild-type D3 (wt-D3) ES cells and stably transduced ES cells (pStat3-D3) at various time points.

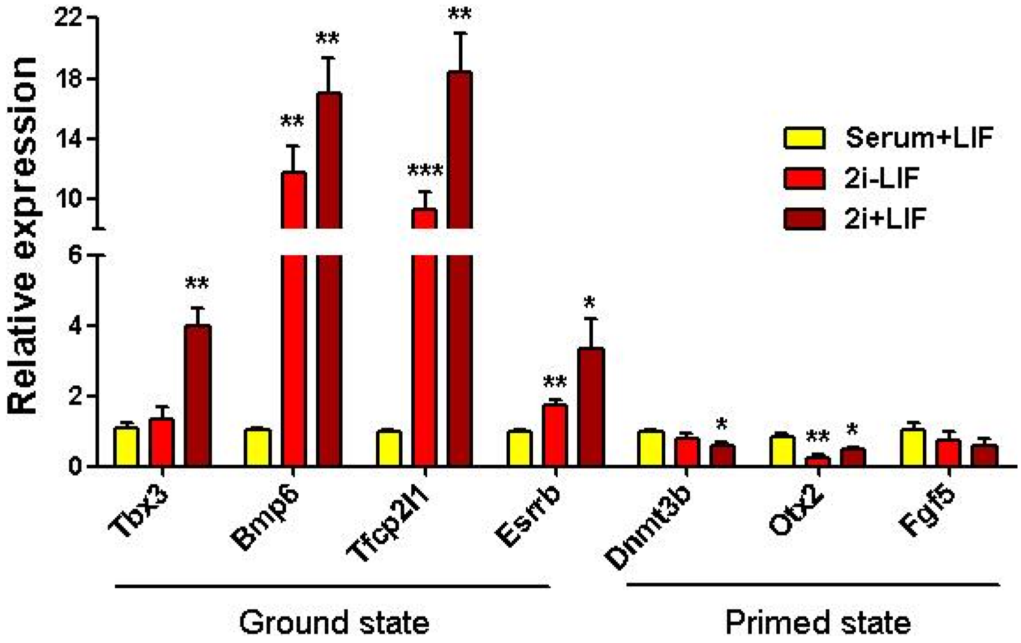

Supplementary Figure 2: Relative expression of ground state and primed state genes in serum+LIF, 2i-LIF and 2i+LIF. Real-time PCR showed the culture conditions of 2i-LIF and 2i+LIF could maintain the ground state of ES cells significantly. ($n = 3$; *$p < .05$, **$p < .01$, ***$p < .001$)

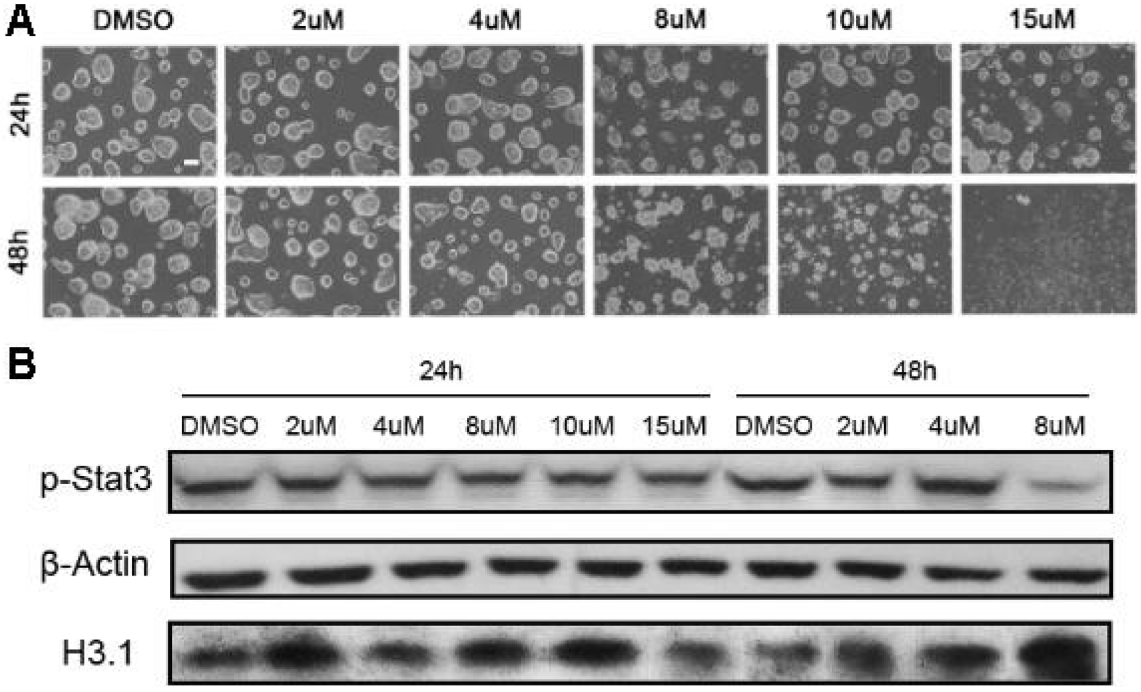

Supplementary Figure 3: pStat3-D3 ES cells with Stattic at different concentrations for 24 hours or 48 hours. (**A**) The cellular morphology of different concentrations and points with Stattic treatment. Scale bars: 100 μm. (**B**) Western blot with total Stat3 and p-Stat3 Y705 antibodies showed Stattic effectively repressed the phosphorylation of Stat3 at 8 μM for 48 hours.

Supplementary Table 1: Primers for qPCR analysis

Gene	Forward primer	Reverse primer
β-Actin	GAGATTACTGCTCTGGCTCCTA	GGACTCATCGTACTCCTGCTTG
Tbx3	GCAGTGGATGTCCAAAGTCGTCACT	CAGGTAGGTTCGAAAAGTACTGTAA
Bmp7	TACGTCAGCTTCCGAGACCT	GGTGGCGTTCATGTAGGAGT
Tfcp2l1	AGGTGCTGACCTCCTGAAGA	GTTTTGCTCCAGCTCCTGAC
Esrrb	CGCCACTCAAGAAGCCATTG	CATGTATGGGCAAAGGGGGA
Dnmt3b	GCTATTTGTCTTGAGGCGCT	GCTATTTGTCTTGAGGCGCT
Otx2	GGAGAGGACGACATTTACTAGG	TTCTGACCTCCATTCTGCTG
Fgf5	TCTTCTGCAGCCACCTGATC	AGTCTGTACTTCACTGGGCTGG
Stat3	GCCATCCTAAGCACAAAGCC	GGGAATGTCGGGGTAGAGGT
Socs3	ATTCACCCAGGTGGCTACAG	GCCAATGTCTTCCCAGTGTT
Nanog	GCTCCGCTCCATAACTTCG	ACCTGGCTTTGCCCTGACT
